# Supplementary material for: COVID-19 vaccine acceptance, hesitancy, and associated factors among medical students in Sudan
Source: PLoS One. 2022 Apr 7;17(4):e0266670. doi: 10.1371/journal.pone.0266670 (PMC8989287; doi:10.1371/journal.pone.0266670)
Supplement: Attachment — Submitted filename: Response to reviewers.docx [file pone.0266670.s002.docx]

PONE-D-21-29953

**COVID-19 vaccine acceptance, hesitancy, and associated factors among medical students in Sudan**

PLOS ONE

| **Reviewers' comments** | **Authors’ reply** |
| --- | --- |
| **Reviewer 1 (Minor comments)** | |
| 1) The statistical technique used in this study is commendable. However, could the authors explain how his results are significant with a small sample size? Also, the study was conducted at faculty that can accommodate more than 1000 medical students. So, what are the reasons primary medical students are not included? | Thank you for commending our statistical techniques. Regarding the sample size, the minimum required sample size for the selected study population was calculated with an appropriate formula and the number of participants was successfully attained. Therefore, we believe the sample size is reasonably representative at least to the medical students whom the sample was drawn from. The faculty can indeed accommodate more than 1000 medical students in all their academic years. However, we selected those in their clinical years for the following reasons. First, due to the COVID-19 partial lockdown at the inception of the study, there were interruptions in the academic program and those in their clinical years were thought to be more suitable for inclusion due to their hospital attachments and relatively uninterrupted schedule. Second, the objective of the study was to conduct an online, simple random design survey. Clinical-year students were more active online in their Telegram groups for the sake of coordinating their clinical attachments. Therefore, they were more appropriate for sampling and more likely to meet the inclusion criteria. |
| 2) The Manuscript concluded that there is a high hesitancy against the COVID-19 vaccine among medical students. However, the medical students are highly educated among whole population. Therefore, could authors explain the reasons behind hesitation among medical students in the discussion, which are different from similar studies mentioned in the manuscript. Also, is this hesitation present in the general population? | A detailed discussion of the reasons behind hesitancy among the medical students has been added in lines 264 through 277. Unfortunately, no published study of COVID-19 hesitancy in the general population exists so far to compare our findings. We have mentioned that in the discussion and recommended such a study in the future. |
| 3) There are issues in the reference section. Please correct it accordingly. | References have been revised and issues resolved. |
| **Reviewer 2 (Suggestions)** | |
| 1. What % of COVID-19 vaccine hesitancy is there in Sudan among whole population? | Unfortunately, no published report currently exists in the literature regarding the percentage of COVID-19 vaccine hesitancy among the general population in Sudan. Investigating the degree of vaccine hesitancy in the whole population was beyond the scope of the study. This has now been mentioned in the discussion section and a recommendation made for a large-scale population study. |
| 2. The authors may additionally provide one Figure as summary, challenges, or prospect of the present study. | A new figure (Fig 2) has been added as suggested. |
| 2. The authors should cross-check all abbreviations in the manuscript. Initially, define in full name followed by abbreviation. | Abbreviations have been cross-checked and all issues corrected. |
| 3. The English of manuscript can be polished (minor). | The manuscript's English has been revised and polished both manually and with the help of the *Grammarly* app. |
| 4. Authors should add a paragraph to discuss more about the cause of COVID-19 vaccine hesitancy among medical students in Sudan. | A new paragraph has been added in the discussion section to discuss the cause of COVID-19 vaccine hesitancy in detail (lines 264 - 277). |
